# A nationally quasi-experimental study to assess the impact of partial organized breast and cervical cancer screening programme on participation and inequalities

**DOI:** 10.1186/s12885-020-07686-4

**Published:** 2020-12-04

**Authors:** Heling Bao, Limin Wang, Matthew Brown, Mei Zhang, Katherine Hunt, Jiangli Di, Zhenping Zhao, Shu Cong, Jing Fan, Liwen Fang, Linhong Wang

**Affiliations:** 1 National Center for Chronic and Non-communicable Disease Control and Prevention, Chinese Center for Disease Control and Prevention, 27 Nanwei Road, Xicheng District, Beijing, 100050 China; 2 Center for Global Health, China Office, National Cancer Institute, National Institutes of Health, Bethesda, USA; 3 Susan G. Komen, 5005 LBJ Freeway, Suite 526, Dallas, TX 75244 USA; 4 National Center for Women and Children’s Health, Chinese Center for Disease Control and Prevention, 12 Dahuisi Road, Haidian District, Beijing, 100081 China

**Keywords:** Breast cancer screening, Cervical cancer screening, Participation, Inequality, Impact, Quasi-experimental study

## Abstract

**Background:**

Organized breast and cervical cancer screening programme could only provide services at no cost for a fraction of socioeconomic-deprived women in China and other low-resource countries, however, little evidence exists for whether such a programme effectively affect the participation and inequalities at the population level.

**Methods:**

We used individual-level data from a nationally cross-sectional survey in 2014–2015 for breast and cervical cancer screening participation. Data for intervention and comparison grouping were from 2009 to 2014 national breast and cervical cancer screening programme, and counties covered by the programme were divided into intervention group. We assessed the impact of the intervention on the participation rates and the magnitude of inequalities with two approaches: multivariable multilevel logistic regressions adjusting for individual- and region-level covariates; and a difference analysis combined with propensity score matching that estimated the average intervention effect.

**Results:**

Of 69,875 included women aged 35–64 years, 21,620 were classified into the intervention group and 43,669 into the comparison group for breast cancer screening; and 31,794 into the intervention group, and 33,682 into the comparison group for cervical cancer screening. Participation rate was higher in intervention group than comparison group for breast cancer screening (25.3, 95% confidential interval [CI], 22.8–27.7%, vs 19.1, 17.4–20.7%), and cervical cancer screening (25.7, 23.8–27.7%, vs 21.5, 19.6–23.5%), respectively. Intervention significantly increased the likelihood of participation for both breast cancer and cervical cancer screening in overall women, rural women and urban women, whereas the effect was significantly higher in rural women than urban women. The average intervention effect on the participation rate was an increase of 7.5% (6.7–8.2%) for breast cancer screening, and 6.8% (6.1–7.5%) for cervical cancer screening, respectively. The inequalities were significantly decreased by 37–41% (*P* < 0.001) between rural and urban, however, were slightly decreased or even increased in terms of age, education status, and household income.

**Conclusions:**

Organized breast and cervical cancer screening programme targeting for a fraction of women could increase the participation rates at population level, however, it could not significantly affect socioeconomic-introduced inequalities. Further studies are need to conduct time-series analyses and strengthen the causal correlation.

## Background

Breast cancer and cervical cancer are important contributors to the female cancer worldwide [1, 2]. Screening for breast cancer could direct early detection and treatment, and reduce the death [3], whereas cervical cancer screening could detect cervical precancerous lesions, stop the occurrence of invasive cancer, and further decrease the cervical cancer incidence [4]. Nonetheless, morbidity and mortality of breast and cervical cancer decrease less or remain unchanged in low-resource settings and lead to widening disparities [5].

Population-based organized breast or cervical cancer screening programme with universal coverage could effectively increase the participation [6, 7]. In low-resource settings, organized programme could only benefit a fraction of women who were difficult to access to services, such as socioeconomic-deprived women [8–10], and therefore would not gain equivalent coverage as that in developed countries. Previous studies showed that the removal of out-of-pocket costs for breast and cervical cancer screening could increase the participation rate [10], but increase the magnitude of inequalities in terms of demographic or socioeconomic status [11]. However, the impact of such a programme for a fraction of eligible women on the participation rates and inequalities is less studied. The absence of evidence is a major obstacle to the implementation and evaluation of such programme in low-resource settings.

The incidence of breast and cervical cancer are substantial in China [2]. However, there was no organized screening programme for breast cancer before 2008 [12]; and cervical cancer screening programme was once conducted at the workplace [13, 14] but broke down following the reforms of the health-care system in the early 1980s [15]. It was estimated that the participation rate for breast or cervical cancer screening was less than 30% in Chinese women aged 20 years or older [16]. In 2009, the Chinese government initiated a national breast and cervical cancer screening programme for women living in rural areas [14]. Because there were many age-eligible women in rural China, the government budget could only afford cost-removing screening for a fraction of women in the programme counties. Specifically, the programme selected counties across China and recruited a fraction of rural women aged 35–64 years. Thus, the initiate of the programme allowed for a comparison of participation rates between women who were in programme counties and those who were not in, and that between rural and urban areas.

In the study, we used a nationally representative, quasi-experimental study to assessed how the organized breast and cervical cancer screening programme for a fraction of eligible women affected the participation rates and socioeconomic inequalities at population level. Furthermore, we estimated the average intervention effect attributed to the programme.

## Methods

### Study design and participant

We used the individual-level data from the 2014–2015 Chinese chronic disease and risk factor survey to measure the participation rates of breast and cervical cancer screening among women aged 35–64 years. We designed a quasi-experimental study by combining the nationally representative survey with the data from the 2009–2014 breast and cervical cancer screening programme. Sampled counties in the survey were divided into intervention group and comparison group with no randomization, according to whether they were covered by the programme. Women in the survey were categorized as rural women (target) and urban women (non-target) based on the place of residence. We compared the difference in screening participation rates between intervention group and control group for overall, rural women, and urban women, respectively. Multivariable multilevel regressions were used to assess the impact of the programme on the participation rates for breast and cervical cancer screening. Difference analyses combined with propensity score matching were used to estimate the average intervention effect. Propensity score matching is increasingly used to balance the bias of covariates in the evaluation of intervention when randomization is not feasible in observational studies [17]. Additionally, we analyzed the change of the relative and absolute inequalities in terms of demographic and socioeconomic status.

This study was approved by the ethics committees of the National Center for Chronic and Non-Communicable Disease Control and Prevention. All participants provided written informed consent before any study procedures.

### Procedure

During 2009–2014, the organized screening programme covered 714 counties for breast cancer screening and 1306 counties for cervical cancer screening across China. County maternal and child health care center in the programme enrolled a fraction of rural women aged 35–64 years (the age range was 30–59 years during 2009–2011 and then switched to 35–64 years since 2012), and provided them with breast cancer screening, or cervical cancer screening, or both at no cost. According to the National Statistics Census [18], rural areas were defined as villages and townships in a county, whereas urban areas were defined as towns, suburbs, or central areas. Additionally, women who were not included in the organized programme could also access to self-supporting screening in health check-up or clinic visiting. During 2009–2014, the programme screened 4.8 million rural women for breast cancer, and screened 40 million for cervical cancer nationwide.

The national cross-sectional survey was described in greater detail previously [19]. Briefly, the survey applied a multistage, systematic, clustered sampling in which 297 counties were randomly selected to create a nationally representative sample. Participants (*n* = 179,347) aged 18 years or older were enrolled, and characteristics of demo-graphic, risk factors, or behaviors related to chronic diseases were collected through a face-to-face interview. All 69,875 female participants aged 35–64 years were abstracted for this study. Considering the birth cohort effect, those women were 30–59 years in 2009 which was also eligible for the programme.

We linked the 297 sampled counties in the survey with the counties in the programme by unique county code and name. The sampled counties overlapped with the programme counties were classified into intervention group, whereas other counties were classified into comparison group. For breast cancer screening, 99 sampled counties were in the intervention group and 198 were in the comparison group; for cervical cancer screening, 142 were in the intervention group and 155 were in the comparison group. There were 90 sampled counties covered by both breast and cervical cancer screening programme (see Additional file 1 Fig. S1). Women in the survey were also categorized as rural women or urban women according to the definition concordant with the programme. Rural women in the intervention group were eligible for cost-removing screening; by contrast, urban women in the intervention group and all women in the comparison group were ineligible in the programme.

### Outcome and covariate measurement

The primary outcomes were participation rates of breast and cervical cancer screening. Women who reported receiving breast or cervical cancer screening at least once during 2009–2014 were defined as screening participation. Participation rate was calculated from the number of women participated in screening divided by all women.

Individual-level covariates in the study included: age group; nationality (Han/others); marital status (never/married/others); education attainment (primary school and lower/junior/senior or higher); employment status (not working/ non-agricultural employment/agriculture employment); medical insurance status (no insurance/insurance for employed resident/insurance for unemployed resident); household income (separated by quartiles and don’t know); health checkup (less than 1 year/every 2–3 years/more than 3 years); self-rated health (good or very good/fair/bad or very bad). We also combined the data from national statistics census to collected county-level covariates, including: the proportion of residents residing in urban areas (urbanization), the proportion of residents ≥25 years who are college graduates (education status), the number of health worker per 1000 residents (health care), and the per capita gross domestic product (GDP). County-level variables were categorized into tertiles.

### Statistical analysis

The participation rates were estimated for breast and cervical cancer screening, respectively, and 95% confidence intervals (CI) were estimated accounting for complex survey design. Rao-scott χ^2^ test was used to compare the difference for categorical variables. We estimated the participation rates for rural and urban women in intervention group or comparison group, respectively, and compared them to show the direct and indirect effect introduced by the programme. We used multivariable multilevel logistic regressions with random intercepts at county- and province- levels, adjusting for individual- and county-level covariates, to estimate the effect of the intervention on the likelihood of participation. In these models, we added intervention term alone, intervention term restricting to rural women, and an interaction term of intervention and residency in rural, respectively, to show different effect of the intervention for overall, rural, and urban women, respectively.

Individual matching is an alternative approach to improve balance of covariates in observational studies, especially when the sample size is large enough [17]. In this study, individuals in the intervention group were matched in a 1:1 ratio to the comparison group based on propensity scores, which was calculated from logistic regressions including individual covariates. We conducted the matching for each subgroup of interest using greedy matching method [20]. Then, we calculated the average intervention effects using methods as described by Farzadfar and colleagues [21]. Briefly, we calculated the differences in participation rates between the intervention group and comparison group on the balanced dataset, taking account of the subclassification of residency in place, age group, household income, and education attainment.

Relative index of inequality (RII) was the ratio between the estimated participation rate among women with the highest level (e.g., age, income, or education) and the lowest level, whereas slope index of inequality (SII) measured the absolute difference between the highest level and lowest level [22]. We estimated RII and SII in terms of rural-urban, age, household income, and education by use of generalized linear models, and used an interaction term of socioeconomic variable and intervention to test the significance.

All analyses were done separately for breast and cervical cancer screening. Probability values for statistical tests were two tailed with *P* < 0.05 as statistically significant. Multilevel logistic regressions were estimated with MLwiN (version 2.30), and other analyses were done with SAS software (version 9.4).

## Results

**Fig. 1:**
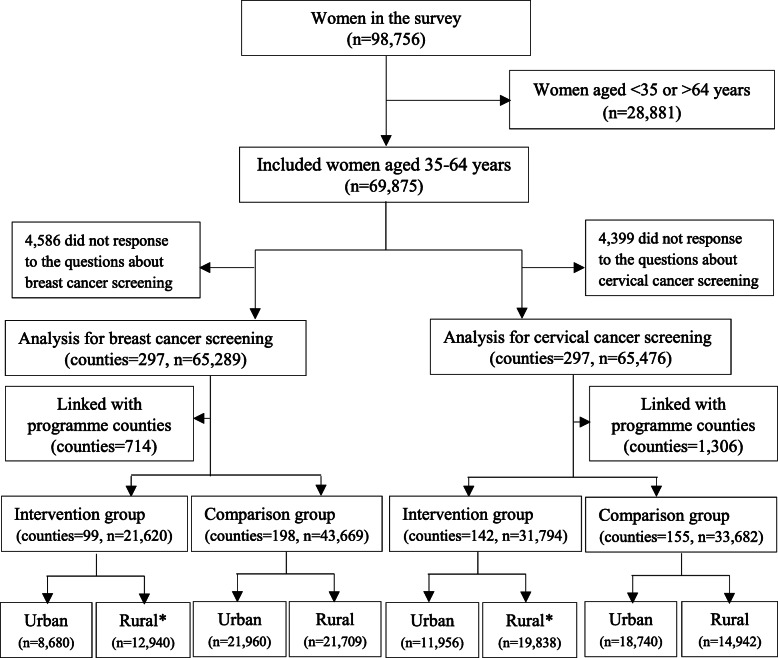
Flowchart of eligible participants and grouping in the study. Notes: * In these rural areas, the programme provided cost-removal breast and cervical cancer screening to women aged 35–64 years

Of 69,875 included women in the survey, 65,289 were included in the analysis for breast cancer screening and 65,476 women were included for cervical cancer screening. For breast cancer screening, 21,620 women were divided into the intervention group and 43,669 were into the comparison group; for cervical cancer screening, 31,794 women were divided into the intervention group and 33,682 were into the comparison group (Fig. 1). There were significant differences in the distribution of some socio-demographic characteristics between the intervention and comparison groups (see Additional file 1 Table S1).

**Fig. 2:**
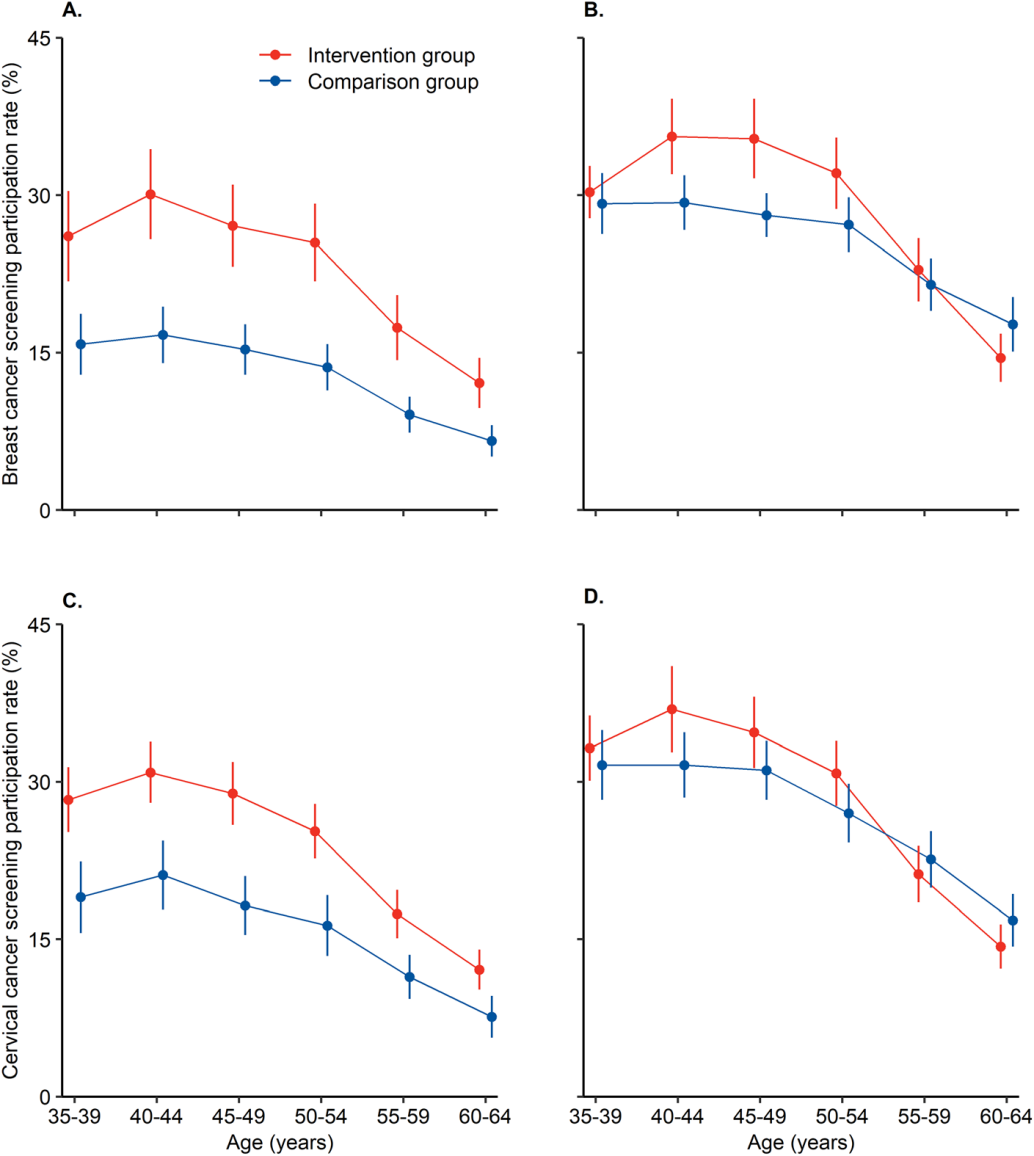
The age-specific participation rates of breast and cervical screening in the intervention and comparison groups by place of residence. **a** breast cancer screening participation rates in rural women. **b** breast cancer screening participation rates in urban women. **c** cervical cancer screening participation rates in rural women. **d** cervical cancer screening participation rates in urban women. Note: 95%CI was estimated by Taylor series variances estimation approach accounting for complex sampling design

Table 1 pt?>showed that participation rates of breast and cervical cancer screening in the intervention group were significantly higher than the comparison group (*P* < 0.001 for both). Participation rates of the intervention group were significantly higher than the comparison group in rural women, however, the differences were not significant in urban women. Figure 2 showed the age-specific participation rates of rural women in the intervention group were significantly higher than rural women in the comparison group, whereas there were not similar patterns for urban women between intervention and comparison group. Additionally, participation rates in women aged 40–54 years, lower education level, and lower household income in the intervention group were significantly higher than the corresponding women in the comparison group (see Additional file 1 Table S2).

**Table 1:** The breast and cervical cancer screening participation rates in intervention and comparison groups by characteristics

	Breast cancer screening			Cervical cancer screening		
	n (%)	Intervention % (95%CI)	Comparison % (95%CI)	n (%)	Intervention % (95%CI)	Comparison % (95%CI)
Overall	65,289 (100.0)	25.3 (22.8–27.7)	19.1 (17.4–20.7)	65,476 (100.0)	25.7 (23.8–27.7)	21.5 (19.6–23.5)
Place of residence						
Rural areas	34,649 (53.1)	23.2 (19.9–26.4)	12.9 (11.0–14.8)	34,780 (53.1)	25.7 (23.5–27.9)	16.9 (14.6–19.3)
Urban areas	30,640 (46.9)	28.4 (25.8–31.0)	25.2 (23.2–27.1)	30,696 (46.9)	30.8 (28.3–33.3)	28.9 (26.6–31.1)
Age group						
35–39	6941 (10.6)	29.6 (26.7–32.5)	24.0 (21.5–26.5)	6963 (10.6)	31.8 (29.5–34.1)	27.7 (24.9–30.4)
40–44	10,819 (16.6)	33.8 (30.5–37.0)	24.1 (21.9–26.3)	10,837 (16.6)	35.0 (32.3–37.6)	28.1 (25.6–30.7)
45–49	13,373 (20.5)	31.9 (28.8–34.9)	22.7 (20.7–24.7)	13,439 (20.5)	33.1 (30.6–35.6)	26.4 (23.9–29.0)
50–54	11,536 (17.7)	29.8 (26.9–32.8)	22.3 (20.3–24.3)	11,570 (17.7)	29.4 (27.1–31.7)	24.5 (22.2–26.7)
55–59	12,425 (19.0)	21.6 (19.2–24.1)	17.5 (15.6–19.4)	12,468 (19.0)	21.0 (19.1–22.9)	20.9 (18.6–23.1)
60–64	10,195 (15.6)	14.1 (12.3–16.0)	14.1 (12.3–15.9)	10,199 (15.6)	14.5 (12.9–16.1)	15.2 (13.3–17.2)
Education attainment						
Primary school and lower	12,481 (19.1)	16.9 (13.8–20.0)	9.0 (7.1–10.9)	12,569 (19.2)	18.3 (16.1–20.4)	11.7 (9.4–13.9)
Junior school	41,724 (63.9)	26.7 (24.2–29.3)	18.7 (16.9–20.4)	41,783 (63.8)	28.0 (25.9–30.1)	21.2 (19.1–23.3)
Senior school and higher	11,047 (16.9)	44.4 (41.1–47.7)	38.2 (35.6–40.8)	11,088 (16.9)	44.1 (41.2–47.0)	38.7 (35.7–41.8)
Annual household income						
1st (lowest) quartile	13,010 (20.0)	20.9 (19.5–23.2)	13.6 (11.5–15.7)	13,062 (20.0)	23.4 (21.3–25.6)	15.8 (13.5–18.0)
2nd quartile	13,837 (21.2)	27.9 (24.8–31.1)	19.2 (17.2–21.3)	13,918 (21.3)	29.0 (26.5–31.5)	21.8 (19.4–24.2)
3th quartile	12,076 (18.5)	32.4 (28.5–36.3)	23.4 (21.2–25.6)	12,122 (18.6)	31.9 (28.7–35.2)	25.8 (23.4–28.3)
4th (highest) quartile	11,416 (17.5)	36.9 (32.7–41.1)	30.5 (27.2–33.8)	11,381 (17.4)	35.7 (31.8–39.7)	33.6 (30.0–37.2)
Refused/don’t know	14,820 (22.7)	20.4 (18.1–22.8)	17.6 (15.8–19.5)	14,863 (22.7)	22.2 (20.2–24.1)	20.0 (17.9–22.1)

Note: 95%CI was estimated by Taylor series variances estimation approach accounting for complex sampling design

*Abbreviations*: *CI* Confidential interval

Table 2 showed the intervention significantly increased the likelihood of participation for breast cancer screening (OR = 1.63, 95%CI 1.56–1.71) and cervical cancer screening (OR = 1.74, 95%CI 1.38–2.20). The interaction term showed the intervention had significantly higher effect in rural women than in urban women (*P* < 0.05 for both); nonetheless, the intervention effect was also significant in urban women. The combination intervention of breast and cervical cancer in the programme had significantly higher effect for either breast cancer or cervical cancer screening than separate intervention. The lower likelihood of screening participation rates was significantly associated with demographic or socioeconomic status in terms of older, lower education attainment, and lower household income (see Additional file 1 Table S3).

**Table 2:** Results of multivariable multilevel logistic regressions for the intervention effect

	Breast cancer screening		Cervical cancer screening	
	Adjusted OR (95%CI)	*P* value	Adjusted OR (95%CI)	*P* value
**Model 1** ^a*^				
Intervention vs. comparison	1.63 (1.56–1.71)	< 0.001	1.74 (1.38–2.20)	< 0.001
**Model 2** ^b*^				
Intervention vs. comparison (rural areas)	1.77 (1.40–2.26)	< 0.001	1.84 (1.45–2.33)	< 0.001
**Model 3** ^c*^				
Intervention vs. comparison (urban areas)	1.54 (1.18–2.00)	0.002	1.63 (1.29–2.07)	< 0.001
Intervention interaction with rural areas	1.31 (1.17–1.47)	< 0.001	1.13 (1.01–1.25)	0.036
**Model 4** ^d*^				
Intervention for one cancer vs. comparison	1.20 (1.07–1.36)	0.002	1.52 (1.12–2.06)	0.007
Intervention for both cancer vs. comparison	1.69 (1.61–1.77)	< 0.001	1.90 (1.46–2.47)	< 0.001

*Abbreviations*: *OR* Odds ratio, *CI* Confidential interval

^*^ All models were adjusting for covariates, including: individual-level age, education attainment, household income, employment status, health insurance, health checkup, self-rated health, and region-level per capita gross domestic product, education status, urbanization, and density of health care worker, with random effect in levels of county and province. All the covariates in the model were assessed using variance influence factor, tolerance, and characteristic root to avoid collinearity

Note: ^a^ Including intervention alone

^b^ Including the crossed classification of intervention and residency in rural, and then comparing the rural women in the intervention group to the rural women in the comparison

^c^ Including the interaction term of intervention and residence. Intervention term showed the intervention effect in urban women; the interaction term of intervention and residency in rural showed the differential effect of intervention in rural women compared with that in urban women

^d^ Intervention group was further divided into intervention for one cancer screening alone and for both breast and cervical cancer screening

**Fig. 3:**
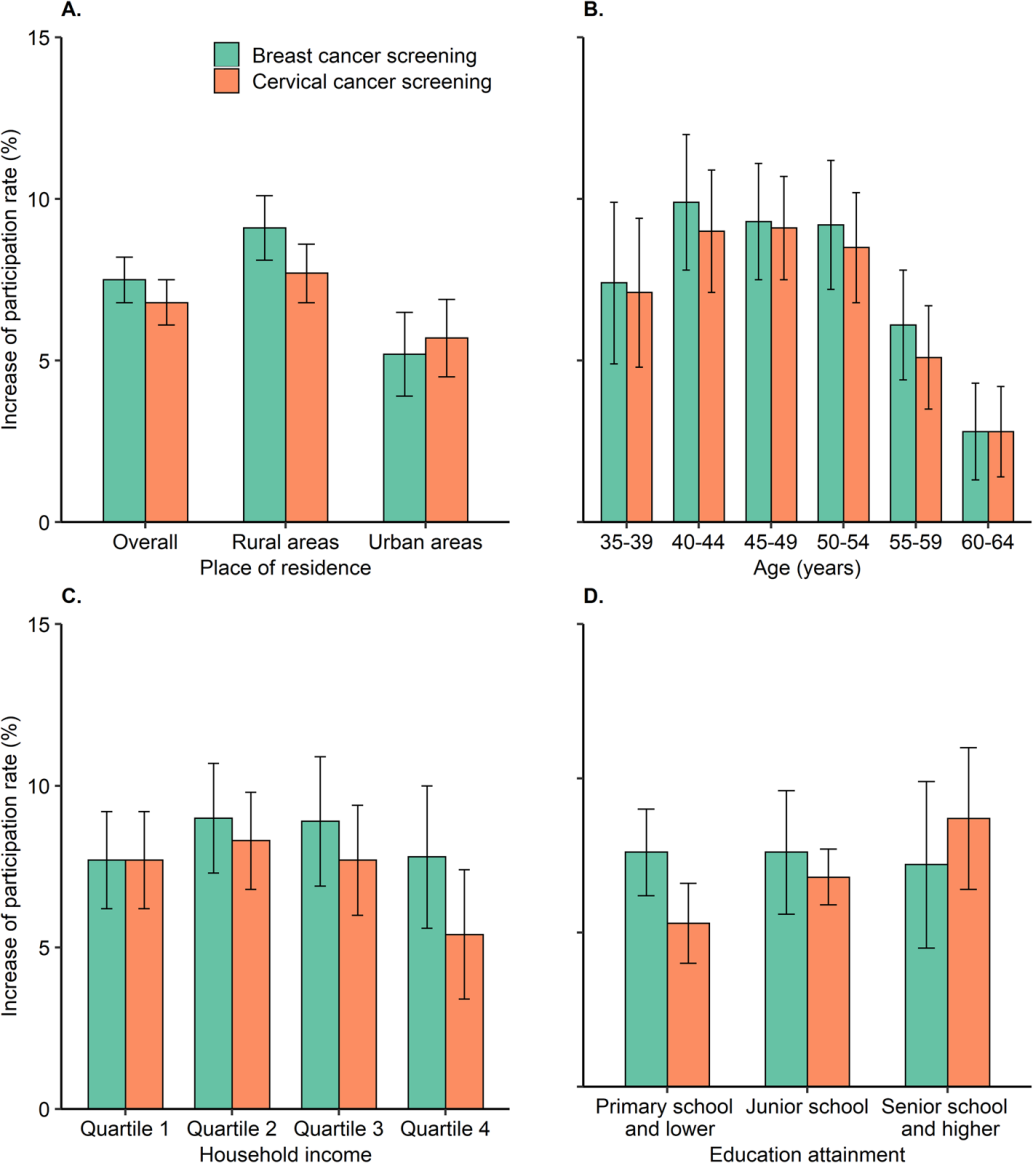
The average intervention effect on the participation rates of breast and cervical cancer screening. **a** intervention effect in overall, rural, and urban women, respectively. **b** intervention effect stratified by age group. **c** intervention effect stratified by household income. **d** intervention effect stratified by education attainment. Note: The average intervention effect was calculated from the difference of participation rates between intervention and comparison group on subclassifications combined with propensity score matching

After matching, the distribution of main demographic factors was not significantly different between the two groups (see Additional file 1 Table S4). The post-matching difference analyses showed that, intervention increased participation rate of breast cancer screening by 7.5% (95%CI 6.7–8.2%), and cervical cancer screening by 6.8% (95%CI 6.1–7.5%) (Fig. 3 and Additional file 1 Table S5). Consistent with findings of intervention effect by multilevel regressions, the average intervention effect was higher in rural women compared with urban women. By contrast, the average intervention effects were relatively lower in older, lower household income, and lower education level subgroups. (see Additional file 1 Table S6).

Table 3 showed that intervention substantially decreased the magnitude of inequalities on participation rates between urban and rural women for both breast and cervical cancer screening, and the relative and absolute indicators significantly decreased ranging from − 37.1% to − 41.9%. Although the relative inequality indicator in terms of household income and education attainment had significantly decreased, absolute indicators changed with no significance. By contrast, relative and absolute inequality indicators in terms of age group substantially increased ranging from 23.1 to 76.9%.

**Table 3:** Estimates and percent change of relative and absolute inequality indicators for cervical and breast cancer screening participation

	Breast cancer screening				Cervical cancer screening			
	Intervention group	Comparison group	Percent change, % ^a^	*P* value for change ^b^	Intervention group	Comparison group	Percent change, % ^a^	*P* value for change ^b^
Rural-urban								
RII (95%CI) ^c^	1.61 (1.39–1.86)	2.72 (2.28–3.24)	−40.8	< 0.001	1.51 (1.32–1.72)	2.40 (2.04–2.83)	−37.1	< 0.001
SII (95%CI) ^c^	9.2 (6.2–12.2)	15.0 (12.3–17.7)	−38.7	0.01	7.5 (4.8–10.1)	12.9 (10.4–15.3)	− 41.9	0.009
Age group								
RII (95%CI)	8.32 (6.35–10.90)	5.24 (3.81–7.21)	58.8	0.03	8.0 (6.2–10.2)	6.5 (4.7–8.8)	23.1	0.29
SII (95%CI)	42.1 (37.0–47.3)	23.8 (19.3–28.3)	76.9	< 0.001	40.4 (35.7–45.0)	26.8 (22.5–31.1)	50.7	< 0.001
Household income								
RII (95%CI) ^c^	1.95 (1.70–2.25)	2.32 (1.95–2.76)	−15.9	0.20	1.52 (1.34–1.73)	2.22 (1.89–2.60)	−31.5	< 0.001
SII (95%CI) ^c^	14.4 (11.3–17.5)	13.2 (10.6–15.8)	9.1	0.17	8.9 (6.2–11.7)	12.1 (9.7–14.5)	−26.4	0.28
Education attainment								
RII (95%CI) ^c^	2.40 (2.08–2.78)	4.08 (3.42–4.86)	−41.2	< 0.001	2.50 (2.19–2.85)	3.42 (2.90–4.03)	−26.9	0.01
SII (95%CI) ^c^	15.4 (12.6–18.2)	17.7 (15.3–20.0)	−13.0	0.66	16.0 (13.5–18.5)	15.2 (13.0–17.4)	5.3	0.05

*Abbreviations*: *RII* Relative index of inequalities, *SII* Slope index of inequalities

^a^ Percent change was calculated from the difference between intervention and comparison divided by the comparison

^b^ *P* values were calculated from generalized linear model including the interaction term of intervention and indicators

^c^ These indicators were calculated adjusting for age group

## Discussion

In the nationally representative analysis, the organized programme targeting for a fraction of rural women significantly increased the participation rates of breast and cervical screening for not only rural women but also urban women. The average intervention effects in participation rates of breast and cervical cancer screening was 7.5% for breast cancer screening and 6.8% for cervical cancer screening, respectively. The intervention effect was significantly higher in rural women than urban women. Using the results of post-matching estimates according to age, the absolute total number of women aged 35–64 years who participated in breast and cervical cancer screening as a result of the programme during 2009–2014 was estimated to be 21.9 million and 20.4 million women, respectively. For breast cancer, the detected cases in early stage due to increase in intervention was estimated as 15,987 according to detection rate of breast cancer at 0–3 stage [23]. Based on the detection rate of cervical intraepithelial lesions grade 2/3 or adenocarcinoma in situ [24], the detected cases with cervical precancerous lesions was estimated as 23,063 cases, who had higher risk of progression to invasive cancer.

The present study showed that participation rates of urban women who were ineligible for the cost-removing screening in the intervention group also significantly increased during the period. This finding indicates the indirect effect of the organized programme on the ineligible women, consistent with previous studies [10, 11]. Yutaka et al. found that free-screening for women with targeted age might positively affect the screening rates of age-ineligible women, however, they did not give the estimation of the indirect effect [10]. In our study, we conducted the analyses restricting to urban women (ineligible for cost-removing screening) in the intervention group or comparison group, to estimate the indirect effect. Individual matching balanced the distribution of characteristics that may affected the screening. The reason behind the indirect effect would be explained as raising awareness, providing education, addressing barriers, motivating women, and peer pressure from the programme [10, 25]. Furthermore, the implementation of the programme based on the maternal and child health care network may also help to remove structural, financial, and technical barriers and improve the availability and accessibility of cancer prevention in programme counties [26, 27].

On the assumption that free-screening (4.8 million breast cancer screening and 40 million cervical cancer screening) were delivered to unscreened women, the average intervention effect should be substantially higher for cervical cancer compared with that for breast cancer screening. However, the average intervention effect was relatively high for breast cancer screening in our results. Many reasons may explain this. Firstly, the proportion of counties implementing the two programme is higher in breast cancer screening programme than that in cervical cancer screening programme (91% vs 63%), and therefore the combination of two screening programme has higher effect on the breast cancer screening compared with cervical cancer screening. Secondly, for cervical cancer, women with better compliance behavior, cervical and/or vaginal symptoms, or history of human papillomavirus infection or abnormal cytology were more likely to attend the programme at a shorter interval. The magnitude of over-screening for cervical cancer needs to be further explored. Thirdly, the outreach modalities might differently affect the participation rate of breast and cervical cancer screening, e.g., group education and small media might be more effective in participation of breast cancer screening than cervical cancer screening [28, 29]. Finally, the questionnaire was not designed for the evaluation, and several screening modalities for breast cancer, such as breast self-examination, might be included in individuals’ responses [11]. Therefore, the average intervention effect may be widened for breast cancer screening but be narrowed for cervical cancer screening.

Our results showed that an organized screening programme targeting for a fraction of women would affect the magnitude of inequalities on screening participation, consistent with previous studies [30, 31]. Intervention targeting for rural women significantly narrowed the rural-urban inequalities, however, the magnitudes in terms of age, household income, and education attainment were slightly decreased or not. Although relative inequalities are decreased in some socioeconomic terms, the absolute indicators that is recommended as primacy show different patterns. Substantial increases in both relative and absolute indicators in terms of age group may decrease the cost-effectiveness of cancer screening, because the mortality and morbidity of breast and cervical cancer are higher among older women than younger [1, 2]. Although intervention with removal of costs positively affected the participation, organized activities co-existing with unrestricted opportunistic screening might consume public resources [11, 31]. That screening register system combining the organized programme with opportunistic screening in routine practice might play a role in reduction of the inequalities, but the effect should be further studied.

Due to the huge expenditure of population-based breast and cervical cancer screening, organized programme serving for a fraction of underserved women at no cost isfeasible in low-resource settings. Although our results show that such programme could positively affect the population-level participation rate for cancer screening, it also has several challenges [32–35]. According to Zhao and et al. [36], if the elimination of cervical cancer would be achieved in China, the coverage of once-in-a-lifetime cervical cancer screening is required to reach 90% in urban and 33% in rural with vaccination of 95% coverage for girls aged 12 years. It demands the programme a larger government-support budget to expanded the screening coverage from the current status in rural and urban areas [19]. Moreover, the cost-effectiveness of the programme should be considered as priority in low-resource settings [37]. Inefficient delivery strategy would widen the disparities within women who were in lower socioeconomic position and at higher risk of developing cancer [38]. Serving women who were rarely or never screened should be a key component [39, 40]. To address these barriers, improved delivery strategies are urgently needed to expand the programme moving forwards, such as timely individual invitation [40, 41], monitoring and management of screening performance [42], and innovation to develop the evidence base for actions [43, 44].

To our knowledge, this is the first study to use nationally representative, cross-sectional study to evaluate the impact of an organized breast and cervical cancer programme serving for a fraction of underserved women on the participation and inequalities in low-resource settings. Large sample size and generalized conclusions are our strengths, which include multilevel data sources, and enable a robust statistical analysis and control for potentially confounding variables.

Some limitations should be discussed. A key limitation is that our results might be affected by selection bias, because no randomization could control for unmeasured or unknown confounders. Although propensity score matching was used to maximum decreased the bias caused by the different distribution of demographic factors, some unmeasured factors would affect the results which should be interpreted with caution. A round of cross-sectional survey may not consider the dynamic changes of programme and other covariates associated with screening participation, and hence, time-series analyses should be conducted to strengthen the causal correlation. Self-reported information may introduce the misclassification bias. Nonetheless, two distinct statistical approaches were applied to control for potential confounding in different ways, and the conclusions about the impact of the intervention were consistent. Finally, the survey could not distinguish whether the self-reported testing was performed for cancer screening or disease diagnosis, but the post-matching estimate for intervention would be not affected because the bias would distributed equally between the intervention and comparison group.

## Conclusions

An organized breast and cervical cancer screening programme targeting for a fraction of rural women could significantly increase the participation rates for both programme-eligible and -ineligible women, and reduce the rural-urban inequalities, however, the magnitude of inequalities in terms of age, household income, and education were slightly decreased or not. The causal correlation of such programme and change of participation rate at the population level needs further time-series study.

## Supplementary Information

**Additional file 1.** Supplementary data.

## Data Availability

The dataset used and/or analyzed during the current study are available from corresponding author on reasonable request.

## Abbreviations

CI: Confidence intervals
RII: Relative index of inequalities
SII: Slope index of inequalities
