# Supplementary material for: A nationally quasi-experimental study to assess the impact of partial organized breast and cervical cancer screening programme on participation and inequalities
Source: BMC Cancer. 2020 Dec 4;20:1191. doi: 10.1186/s12885-020-07686-4 (PMC7716588; doi:10.1186/s12885-020-07686-4)
Supplement: Supplementary file 1 — Additional file 1. Supplementary data. [file 12885_2020_7686_MOESM1_ESM.docx]

**Additional file 1: supplementary data**

**A nationally quasi-experimental study to assess the impact of partial organized breast and cervical cancer screening program on participation and inequalities.**

**Fig. S1** Sample counties in the survey stratified by the coverage of the programme.

**Table S1** The characteristics of women in intervention and comparison groups.

**Table S2** The breast and cervical cancer screening participation rates by characteristics.

**Table S3** The multilevel regressions for breast and cervical cancer screening adjusting for covariates.

**Table S4** The post-matching distribution of characteristics in the intervention and comparison groups.

**Table S5** The pre-matching and post-matching estimates for participation rates of breast and cervical cancer screening in the intervention and comparison, respectively.

**Table S6** The average intervention effect in the participation rates of breast and cervical cancer screening, stratified by place of residence.


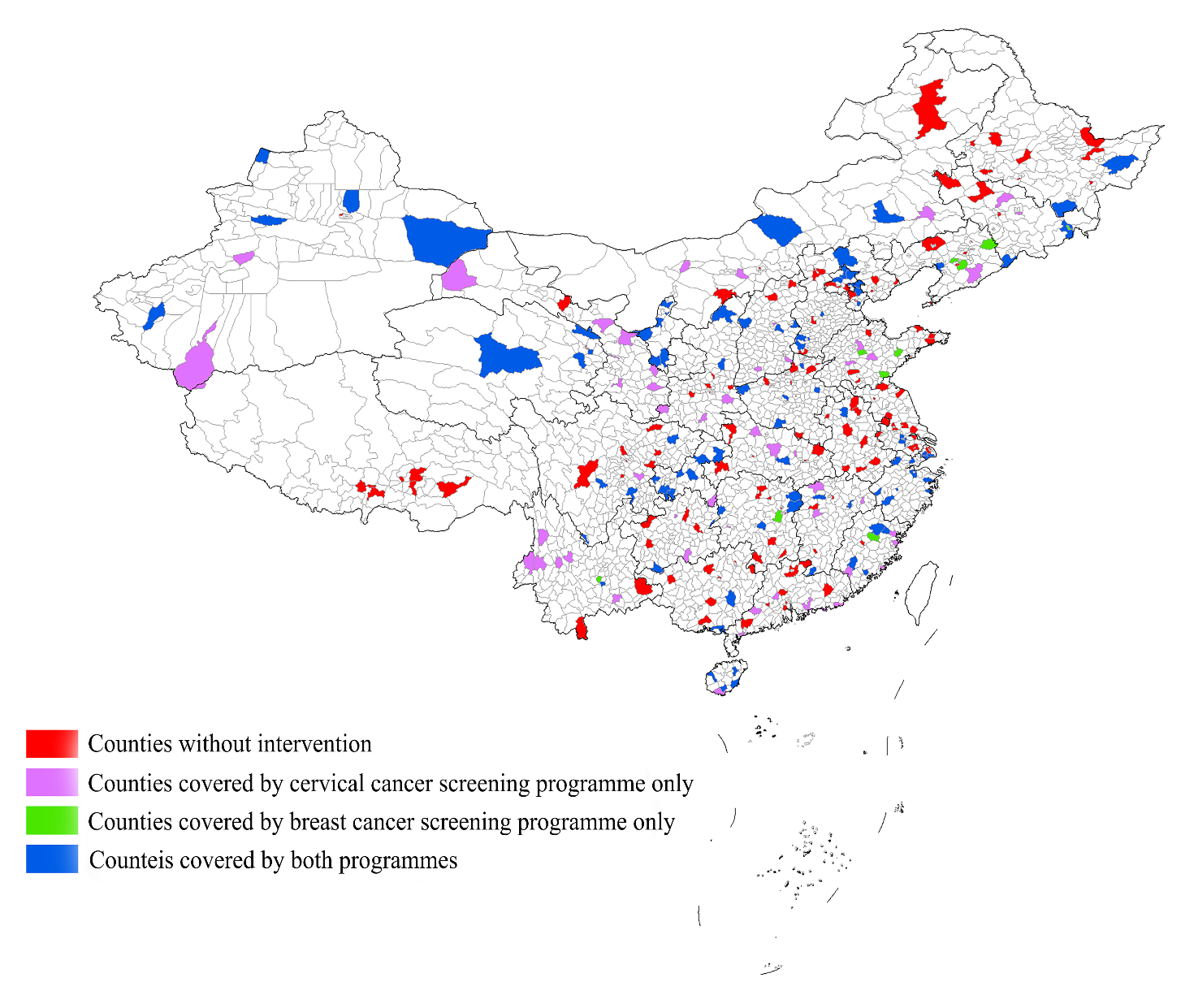


**Fig. S1** Sample counties in the survey stratified by the coverage of the programme.

**Table S1.** The characteristics of women in intervention and comparison groups.

|  | Breast cancer screening (n=65,289) | | |  | Cervical cancer screening (n=65,476) | | |
| --- | --- | --- | --- | --- | --- | --- | --- |
|  | Intervention  (n=21,620) | Comparison  (n=43,669) | *p* value |  | Intervention  (n=31,794) | Comparison  (n=33,682) | *p* value |
| Place of residence |  |  | 0.003 |  |  |  | <0.001 |
| Urban | 40.1 (36.9-43.4) | 50.3 (46.8-53.8) |  |  | 37.6 (34.4-40.8) | 55.6 (51.8-59.5) |  |
| Rural | 59.9 (56.6-63.1) | 49.7 (46.2-53.2) |  |  | 62.4 (59.2-65.6) | 44.4 (40.5-48.2) |  |
| Age group |  |  | 0.499 |  |  |  | 0.002 |
| 35-39 | 10.6 (9.9-11.2) | 10.7 (10.2-11.2) |  |  | 11.0 (10.4-11.6) | 10.3 (9.8-10.8) |  |
| 40-44 | 16.9 (16.4-17.3) | 16.4 (15.9-17.0) |  |  | 17.3 (16.7-17.9) | 15.8 (15.3-16.4) |  |
| 45-59 | 20.9 (20.3-21.5) | 20.3 (19.7-20.9) |  |  | 21.0 (20.4-21.5) | 20.1 (19.4-20.8) |  |
| 50-54 | 17.1 (16.6-17.6) | 18.0 (17.5-18.4) |  |  | 17.5 (17.0-17.9) | 17.9 (17.4-18.4) |  |
| 55-59 | 18.7 (18.1-19.2) | 19.2 (18.6-19.8) |  |  | 18.4 (17.8-18.9) | 19.7 (19.0-20.3) |  |
| 60-64 | 15.9 (15.3-16.5) | 15.5 (14.8-16.1) |  |  | 14.9 (14.3-15.5) | 16.2 (15.5-16.9) |  |
| Education attainment |  |  | <0.001 |  |  |  | <0.001 |
| Primary school and lower | 20.5 (18.8-22.2) | 18.4 (16.6-20.3) |  |  | 22.4 (20.4-24.4) | 16.2 (14.5-17.9) |  |
| Junior school | 67.3 (65.7-68.9) | 62.3 (60.4-64.2) |  |  | 66.5 (64.6-68.4) | 61.3 (59.2-63.5) |  |
| Senior school and higher | 12.2 (11.0-13.5) | 19.3 (17.4-21.2) |  |  | 11.1 (9.7-12.6) | 22.4 (20.2-24.7) |  |
| Employment status |  |  | 0.026 |  |  |  | <0.001 |
| Non-agriculture employment | 16.8 (15.3-18.3) | 21.4 (19.5-23.3) |  |  | 15.6 (13.9-17.3) | 23.8 (21.7-26.0) |  |
| Agricultural employment | 51.1 (47.9-54.2) | 44.6 (41.3-27.9) |  |  | 54.8 (51.6-58.1) | 39.3 (35.7-42.8) |  |
| Not working | 32.1 (29.6-34.6) | 34.0 (31.6-36.3) |  |  | 29.6 (26.9-32.3) | 36.9 (34.5-39.3) |  |
| Household income |  |  | 0.214 |  |  |  | <0.001 |
| 1st (lowest) quartile | 21.5 (19.4-23.6) | 19.2 (17.5-20.9) |  |  | 23.3 (21.4-25.2) | 16.8 (15.1-18.5) |  |
| 2nd quartile | 22.3 (21.0-23.5) | 20.7 (19.7-21.8) |  |  | 22.9 (21.8-24.1) | 19.8 (18.6 -20.9) |  |
| 3th quartile | 17.4 (16.5-18.4) | 19.1 (17.9-20.2) |  |  | 16.7 (15.7-17.8) | 20.3 (19.0-21.5) |  |
| 4th (highest) quartile | 15.7 (14.2-17.2) | 18.4 (16.7-20.1) |  |  | 14.5 (13.1-16.0) | 20.2 (18.2-22.1) |  |
| Refused/Don’t know | 23.1 (20.6-25.6) | 22.6 (20.4-24.8) |  |  | 22.5 (20.3-24.7) | 23.0 (20.6-25.4) |  |
| Health insurance |  |  | <0.001 |  |  |  | <0.001 |
| Insurance for employed resident | 10.3 (9.0-11.7) | 21.1 (18.3-23.9) |  |  | 8.7 (7.0-10.3) | 25.9 (22.5-29.2) |  |
| Insurance for unemployed resident | 87.9 (86.3-89.4) | 76.9 (74.0-79.8) |  |  | 89.8 (88.1-91.6) | 71.7 (68.2-75.3) |  |
| No insurance | 1.8 (1.5-2.1) | 2.0 (1.8-2.3) |  |  | 1.5 (1.2-1.8) | 2.4 (2.1-2.7) |  |
| Frequency of health checkup |  |  | 0.186 |  |  |  | <0.001 |
| Less than 1 year | 25.1 (23.3-26.9) | 22.6 (20.9-24.3) |  |  | 20.6 (19.0-22.3) | 26.0 (23.9-28.2) |  |
| 2-3 years | 8.0 (7.3-8.7) | 7.7 (7.1-8.3) |  |  | 7.0 (6.4-7.7) | 8.4 (7.8-9.1) |  |
| More than 3 years | 4.4 (3.6-5.2) | 5.6 (4.9-6.3) |  |  | 4.5 (3.8-5.2) | 5.9 (5.1-6.6) |  |
| Never | 62.5 (60.4-64.6) | 64.1 (61.8-66.3) |  |  | 67.8 (65.7-69.9) | 59.7 (57.0-62.3) |  |

**Table S2.** The breast and cervical cancer screening participation rates by characteristics.

|  | Urban women | | |  | Rural women | | |
| --- | --- | --- | --- | --- | --- | --- | --- |
|  | Intervention |  | Comparison |  | Intervention |  | Comparison |
| **Breast cancer screening** |  |  |  |  |  |  |  |
| Age group (years) |  |  |  |  |  |  |  |
| 35-39 | 32.8 (30.2-35.5) |  | 30.9 (28.0-33.8) |  | 27.5 (23.4-31.5) |  | 16.8 (13.8-19.8) |
| 40-44 | 37.7 (34.2-41.2) |  | 31.1 (28.6-33.7) |  | 31.1 (26.8-35.5) |  | 17.8 (15.2-20.4) |
| 45-49 | 37.2 (33.3-41.1) |  | 30.0 (27.8-32.1) |  | 28.5 (24.7-32.2) |  | 16.5 (14.2-18.8) |
| 50-54 | 34.4 (30.8-38.0) |  | 29.5 (26.9-32.1) |  | 26.8 (23.2-30.5) |  | 14.6 (12.5-16.8) |
| 55-59 | 26.0 (23.2-28.8) |  | 24.0 (21.4-26.7) |  | 18.6 (15.4-21.8) |  | 9.9 (8.1-11.8) |
| 60-64 | 16.0 (13.6-18.3) |  | 20.2 (17.6-22.8) |  | 12.8 (10.4-15.2) |  | 7.3 (5.8-8.8) |
| Education attainment |  |  |  |  |  |  |  |
| Primary school and lower | 14.6 (11.1-18.0) |  | 12.7 (10.2-15.2) |  | 17.7 (14.5-20.9) |  | 7.6 (5.8-9.5) |
| Junior school | 28.8 (26.3-31.4) |  | 22.7 (20.7-24.7) |  | 25.4 (22.1-28.7) |  | 15.3 (13.2-17.3) |
| Senior school and higher | 45.3 (41.2-49.4) |  | 39.9 (36.9-42.8) |  | 42.1 (38.2-46.0) |  | 27.8 (24.4-31.3) |
| Annual household income |  |  |  |  |  |  |  |
| 1st quartile | 26.9 (23.8-30.0) |  | 19.7 (17.0-22.5) |  | 18.5 (16.0-21.0) |  | 10.9 (8.9-13.0) |
| 2nd quartile | 31.0 (27.7-34.2) |  | 24.9 (22.6-27.3) |  | 26.2 (22.2-30.3) |  | 15.5 (13.0-18.0) |
| 3th quartile | 33.1 (29.3-37.0) |  | 28.7 (26.3-31.1) |  | 31.7 (25.6-37.8) |  | 15.8 (12.9-18.7) |
| 4th quartile | 37.3 (33.2-41.4) |  | 34.5 (30.9-38.1) |  | 36.4 (30.7-42.1) |  | 18.5 (14.0-23.0) |
| **Cervical cancer screening** |  |  |  |  |  |  |  |
| Age group (years) |  |  |  |  |  |  |  |
| 35-39 | 35.2 (32.2-38.1) |  | 33.6 (30.3-36.9) |  | 29.8 (27.0-32.6) |  | 20.0 (16.8-23.2) |
| 40-44 | 39.1 (35.2-43.1) |  | 33.6 (30.7-36.6) |  | 32.5 (29.7-35.3) |  | 22.0 (18.9-25.1) |
| 45-49 | 36.9 (33.3-40.4) |  | 33.1 (30.2-36.0) |  | 30.9 (28.2-33.6) |  | 19.4 (16.7-22.1) |
| 50-54 | 33.0 (29.5-36.4) |  | 29.6 (27.0-32.3) |  | 27.2 (24.6-29.8) |  | 17.7 (14.8-20.6) |
| 55-59 | 24.0 (21.5-26.6) |  | 25.9 (23.1-28.7) |  | 19.1 (16.7-21.5) |  | 13.6 (11.5-15.6) |
| 60-64 | 16.2 (13.9-18.5) |  | 19.9 (17.3-22.4) |  | 13.4 (11.4-15.4) |  | 8.7 (6.7-10.8) |
| Education attainment |  |  |  |  |  |  |  |
| Primary school and lower | 17.4 (14.5-20.2) |  | 15.3 (11.9-18.6) |  | 18.5 (16.3-20.8) |  | 10.2 (8.1-12.2) |
| Junior school | 29.5 (26.8-32.1) |  | 23.8 (21.5-26.1) |  | 27.2 (24.8-29.6) |  | 18.5 (16.1-20.9) |
| Senior school and higher | 44.6 (41.1-48.1) |  | 39.9 (36.6-43.2) |  | 43.0 (38.9-47.0) |  | 29.2 (24.8-33.6) |
| Household income |  |  |  |  |  |  |  |
| 1st (lowest) quartile | 27.6 (24.8-30.4) |  | 20.5 (17.5-23.6) |  | 22.0 (19.8-24.3) |  | 13.2 (11.3-15.2) |
| 2nd quartile | 30.9 (27.9-34.0) |  | 26.6 (24.0-29.2) |  | 28.0 (25.1-30.9) |  | 18.0 (15.3-20.6) |
| 3th quartile | 33.6 (29.8-37.4) |  | 29.6 (27.0-32.2) |  | 30.4 (26.0-34.9) |  | 19.6 (16.0-23.2) |
| 4th (highest) quartile | 35.6 (31.2-40.1) |  | 36.5 (32.6-40.3) |  | 35.9 (31.0-40.7) |  | 22.8 (17.3-28.2) |

**Table S3.** The multilevel regressions for breast and cervical cancer screening adjusting for covariates.

| Independent variables | Model 1: breast cancer screening (N=65,031) | |  | Model 2: cervical cancer screening (N=65,218) | |
| --- | --- | --- | --- | --- | --- |
|  | OR (95%CI) | *P* value |  | OR (95%CI) | *P* value |
| **Individual-level** |  |  |  |  |  |
| **Age group (vs. 60-64)** |  |  |  |  |  |
| 35-39 | 2.92 (2.64-3.23) | <0.001 |  | 3.36 (3.05-3.69) | <0.001 |
| 40-44 | 3.30 (3.01-3.61) | <0.001 |  | 3.77 (3.45-4.11) | <0.001 |
| 45-49 | 2.83 (2.60-3.09) | <0.001 |  | 3.24 (2.98-3.52) | <0.001 |
| 50-54 | 2.38 (2.18-2.59) | <0.001 |  | 2.47 (2.28-2.69) | <0.001 |
| 55-59 | 1.52 (1.39-1.65) | <0.001 |  | 1.62 (1.49-1.76) | <0.001 |
| **Han ethnic (vs. other race/ethnic)** | 1.10 (1.00-1.21) | 0.062 |  | 1.12 (1.02-1.23) | 0.016 |
| **Marital Status (vs. never married)** |  |  |  |  |  |
| Married | 1.93 (1.40-2.66) | <0.001 |  | 1.95 (1.45-2.64) | <0.001 |
| Other | 1.79 (1.29-2.49) | <0.001 |  | 1.78 (1.31-2.43) | <0.001 |
| **Education attainment (vs. primary school and lower)** | | |  |  |  |
| Junior school | 1.39 (1.29-1.50) | <0.001 |  | 1.31 (1.22-1.40) | <0.001 |
| Senior school and higher | 2.03 (1.84-2.24) | <0.001 |  | 1.91 (1.74-2.08) | <0.001 |
| **Employment status (vs. not working)** |  |  |  |  |  |
| Non-agricultural employment | 1.11 (1.04-1.18) | 0.001 |  | 1.16 (1.09-1.23) | <0.001 |
| Agriculture employment | 1.04 (0.98-1.11) | 0.224 |  | 1.09 (1.03-1.16) | 0.006 |
| **Medical insurance status (vs. no insurance)** | |  |  |  |  |
| Insurance for employed resident | 1.83 (1.53-2.20) | <0.001 |  | 1.88 (1.57-2.24) | <0.001 |
| Insurance for unemployed resident | 1.51 (1.27-1.80) | <0.001 |  | 1.65 (1.39-1.96) | <0.001 |
| **Household income (vs. 1st quartile)** |  |  |  |  |  |
| 2nd quartile | 1.16 (1.08-1.25) | <0.001 |  | 1.14 (1.06-1.22) | <0.001 |
| 3th quartile | 1.09 (1.01-1.18) | 0.027 |  | 1.09 (1.01-1.18) | 0.019 |
| 4th quartile | 1.18 (1.09-1.28) | 0.541 |  | 1.17 (1.08-1.27) | <0.001 |
| Refused/Don’t know | 0.98 (0.90-1.06) | 0.352 |  | 0.90 (0.84-0.97) | 0.006 |
| **Frequency of health checkup (vs. never)** |  |  |  |  |  |
| Less than 1 year | 3.53 (3.33-3.75) | <0.001 |  | 3.20 (3.03-3.38) | <0.001 |
| 2-3 years | 3.14 (2.90-3.39) | <0.001 |  | 2.90 (2.69-3.12) | <0.001 |
| More than 3 years | 2.41 (2.20-2.65) | <0.001 |  | 2.30 (2.10-2.52) | <0.001 |
| **Self-rated health (vs. good/very good)** |  |  |  |  |  |
| Fair | 1.00 (0.95-1.05) | 0.956 |  | 1.02 (0.98-1.07) | 0.336 |
| Bad/Very bad | 1.09 (1.01-1.18) | 0.028 |  | 1.10 (1.02-1.19) | 0.010 |
| **County-level** |  |  |  |  |  |
| **Intervention (vs comparison)** ^*^ | 1.54 (1.18-2.00) | 0.002 |  | 1.63 (1.29-2.07) | <0.001 |
| **Rural areas (vs urban areas)** | 0.84 (0.77-0.91) | <0.001 |  | 0.95 (0.87-1.04) | 0.297 |
| **Interaction: intervention × rural** ^⁋^ | 1.31 (1.17-1.47) | <0.001 |  | 1.13 (1.01-1.25) | 0.036 |
| **Urbanization (vs. low)** |  |  |  |  |  |
| Medium | 0.84 (0.61-1.16) | 0.291 |  | 0.88 (0.66-1.17) | 0.382 |
| High | 1.18 (0.74-1.89) | 0.484 |  | 0.94 (0.62-1.44) | 0.779 |
| **Education status (vs. low)** |  |  |  |  |  |
| Medium | 1.67 (1.23-2.26) | 0.001 |  | 1.43 (1.09-1.88) | 0.01 |
| High | 1.82 (1.15-2.89) | 0.012 |  | 1.85 (1.21-2.81) | 0.004 |
| **Density of health care worker (vs. low)** | |  |  |  |  |
| Medium | 1.05 (0.54-2.05) | 0.877 |  | 1.03 (0.54-1.96) | 0.92 |
| High | 0.96 (0.43-2.11) | 0.909 |  | 0.83 (0.39-1.79) | 0.644 |
| **per capita GDP (vs. low)** |  |  |  |  |  |
| Medium | 1.71 (0.89-3.25) | 0.107 |  | 1.53 (0.82-2.83) | 0.183 |
| High | 1.70 (0.77-3.76) | 0.192 |  | 1.53 (0.71-3.29) | 0.277 |
| **County-level variance (Standard error)** | 0.750 (0.071) | <0.001 |  | 0.616 (0.058) | <0.001 |
| **Province-level variance (Standard error)** | 0.373 (0.119) | 0.002 |  | 0.357 (0.110) | 0.001 |

Note: ^*^ intervention means the effect on urban women; ^⁋^ the interaction term means the differential effect of intervention in rural women compared with that in urban women. Abbreviations: OR, odds ratio; CI, confidential interval.

**Table S4.** The distribution of characteristics in the intervention and comparison groups after matching.

|  | Breast cancer screening (n=65,289) | | |  | Cervical cancer screening | | |
| --- | --- | --- | --- | --- | --- | --- | --- |
|  | Intervention  (n=21, 499) | Comparison  (n=21,499) | *p* value |  | Intervention  (n=26,322) | Comparison  (n=26,322) | *P* value |
| Age group |  |  | 0.49 |  |  |  | 0.07 |
| 35-39 | 2276 (10.6) | 2252 (10.5) |  |  | 2765 (10.5) | 2850 (10.8) |  |
| 40-44 | 3625 (16.9) | 3568 (16.6) |  |  | 4443 (16.9) | 4522 (17.2) |  |
| 45-59 | 4501 (20.9) | 4446 (20.7) |  |  | 5576 (21.2) | 5740 (21.8) |  |
| 50-54 | 3676 (17.1) | 3793 (17.6) |  |  | 4576 (17.4) | 4517 (17.2) |  |
| 55-59 | 3997 (18.6) | 4086 (19.0) |  |  | 4830 (18.4) | 4759 (18.1) |  |
| 60-64 | 3424 (15.9) | 3354 (15.6) |  |  | 4132 (15.7) | 3934 (15.0) |  |
| Education attainment |  |  | 0.93 |  |  |  | 0.62 |
| Primary school and lower | 4410 (20.5) | 4431 (20.6) |  |  | 5386 (20.5) | 5317 (20.2) |  |
| Junior school | 14 462 (67.3) | 14 463 (67.3) |  |  | 17 601 (66.9) | 17 707 (67.3) |  |
| Senior school and higher | 2627 (12.2) | 2605 (12.1) |  |  | 3335 (12.7) | 3298 (12.5) |  |
| Employment status |  |  | 0.08 |  |  |  | <0.001 |
| Non-agriculture employment | 3610 (16.8) | 3576 (16.6) |  |  | 4411 (16.8) | 4881 (18.5) |  |
| Agricultural employment | 10 982 (51.1) | 11 206 (52.1) |  |  | 13 473 (51.2) | 12 971 (49.3) |  |
| Not working | 6907 (32.1) | 6717 (31.2) |  |  | 8438 (32.1) | 8470 (32.2) |  |
| Household income |  |  | 0.80 |  |  |  | 0.64 |
| 1st (lowest) quartile | 4623 (21.5) | 4599 (21.4) |  |  | 5318 (20.2) | 5370 (20.4) |  |
| 2nd quartile | 4789 (22.3) | 4733 (22.0) |  |  | 5919 (22.5) | 5864 (22.3) |  |
| 3th quartile | 3749 (17.4) | 3828 (17.8) |  |  | 4940 (18.8) | 5040 (19.2) |  |
| 4th (highest) quartile | 3375 (15.7) | 3334 (15.5) |  |  | 4048 (15.4) | 4061 (15.4) |  |
| Refused/Don’t know | 4963 (23.1) | 5005 (23.3) |  |  | 6097 (23.2) | 5987 (22.8) |  |

**Table S5.** The pre-matching and post-matching estimates for participation rates of breast and cervical cancer screening in the intervention and comparison, respectively.

|  | Pre-matching | |  | Post-matching | |
| --- | --- | --- | --- | --- | --- |
|  | Intervention | Comparison |  | Intervention | Comparison |
| **Breast cancer screening** |  |  |  |  |  |
| Place of residence |  |  |  |  |  |
| Rural women | 23.2 (19.4-26.9) | 12.9 (10.7-15.0) |  | 23.2 (22.5-23.9) | 14.1 (13.5-14.7) |
| Urban women | 28.4 (24.5-32.3) | 25.2 (22.9-27.5) |  | 28.4 (27.5-29.4) | 23.2 (22.3-24.1) |
| Age group |  |  |  |  |  |
| 35-39 | 27.8 (24.3-31.3) | 22.6 (20.0-25.2) |  | 27.8 (26.0-29.7) | 20.4 (18.7-22.0) |
| 40-44 | 32.2 (28.0-36.4) | 22.7 (20.3-25.0) |  | 32.2 (30.7-33.7) | 22.4 (21.0-23.7) |
| 45-49 | 30.3 (26.3-34.2) | 21.2 (18.9-23.4) |  | 30.3 (28.9-31.6) | 20.9 (19.7-22.1) |
| 50-54 | 28.1 (24.1-32.0) | 20.5 (18.2-22.8) |  | 28.1 (26.6-29.5) | 18.9 (17.6-20.1) |
| 55-59 | 19.8 (16.6-23.0) | 15.8 (13.6-17.9) |  | 19.8 (18.5-21.0) | 13.7 (12.6-14.7) |
| 60-64 | 13.2 (10.8-15.6) | 12.4 (10.5-14.4) |  | 13.2 (12.0-14.3) | 10.4 (9.4-11.4) |
| Household income |  |  |  |  |  |
| 1st (lowest) quartile | 19.5 (16.5-22.4) | 12.5 (10.3-14.7) |  | 19.5 (18.3-20.6) | 11.8 (10.9-12.7) |
| 2nd quartile | 26.5 (22.8-30.3) | 17.7 (15.5-19.9) |  | 26.6 (25.3-27.8) | 17.6 (16.5-18.6) |
| 3th quartile | 30.2 (25.3-35.1) | 21.4 (19.0-23.8) |  | 30.2 (28.7-31.7) | 21.3 (20.0-22.6) |
| 4th (highest) quartile | 34.9 (29.8-40.1) | 28.6 (25.1-32.1) |  | 34.4 (32.8-36.1) | 26.7 (25.1-28.2) |
| Education attainment |  |  |  |  |  |
| Primary school and lower | 15.8 (12.4-19.2) | 8.2 (6.3-10.1) |  | 15.8 (14.7-16.9) | 8.2 (7.4-9.0) |
| Junior school | 25.2 (21.8-28.5) | 17.1 (15.2-19.1) |  | 25.2 (24.5-25.9) | 17.6 (17.0-18.2) |
| Senior school and higher | 41.8 (36.8-46.8) | 35.7 (32.7-38.8) |  | 41.9 (40.0-43.8) | 34.7 (32.8-36.5) |
| **Cervical cancer screening** |  |  |  |  |  |
| Place of residence |  |  |  |  |  |
| Rural women | 24.0 (21.4-26.7) | 15.6 (12.7-18.4) |  | 23.2 (22.5-23.9) | 15.6 (15.0-16.2) |
| Urban women | 28.6 (25.5-31.7) | 26.3 (23.8-28.8) |  | 28.7 (27.9-29.7) | 23.1 (22.2-23.9) |
| Age group |  |  |  |  |  |
| 35-39 | 30.2 (27.3-33.0) | 26.1 (23.0-29.1) |  | 30.3 (28.6-32.0) | 23.3 (21.7-24.8) |
| 40-44 | 33.2 (29.9-36.4) | 26.5 (23.6-29.4) |  | 33.1 (31.7-34.5) | 24.1 (22.8-25.3) |
| 45-49 | 31.1 (28.2-33.9) | 24.8 (22.0-27.7) |  | 31.5 (30.3-32.7) | 22.4 (21.3-23.5) |
| 50-54 | 27.3 (24.4-30.3) | 22.3 (19.7-24.9) |  | 27.4 (26.1-28.7) | 18.9 (17.8-20.1) |
| 55-59 | 18.9 (16.6-21.2) | 18.0 (15.5-20.6) |  | 19.3 (18.2-20.4) | 14.2 (13.2-15.2) |
| 60-64 | 12.9 (11.2-14.6) | 13.0 (10.7-15.2) |  | 12.9 (11.8-13.9) | 10.1 (9.1-11.0) |
| Household income |  |  |  |  |  |
| 1st (lowest) quartile | 21.6 (19.2-23.9) | 14.1 (11.4-16.8) |  | 21.4 (20.3-22.5) | 13.7 (12.7-14.6) |
| 2nd quartile | 27.2 (24.2-30.2) | 20.0 (17.4-22.5) |  | 27.0 (25.9-28.2) | 18.8 (17.7-19.8) |
| 3th quartile | 29.9 (26.2-33.6) | 23.6 (21.0-26.3) |  | 29.4 (28.2-30.7) | 21.7 (20.6-22.8) |
| 4th (highest) quartile | 33.6 (29.1-38.1) | 31.2 (27.4-34.9) |  | 32.9 (31.4-34.3) | 27.5 (26.1-28.9) |
| Education attainment |  |  |  |  |  |
| Primary school and lower | 16.6 (14.2-19.1) | 10.5 (8.0-13.1) |  | 15.7 (14.7-16.6) | 10.3 (9.5-11.1) |
| Junior school | 26.1 (23.5-28.6) | 19.3 (16.9-21.6) |  | 25.5 (24.8-26.1) | 18.7 (18.1-19.3) |
| Senior school and higher | 42.1 (38.3-45.9) | 35.8 (32.4-39.2) |  | 42.0 (40.3-43.7) | 33.4 (31.7-35.0) |

**Table S6.** The average intervention effect in the participation rates of breast and cervical cancer screening, stratified by place of residence.

|  | Average intervention effect | |
| --- | --- | --- |
|  | Rural women | Urban women |
| **Breast cancer screening** |  |  |
| Age group |  |  |
| 35-39 | 10.2 (7.1-13.2) | 4.0 (-0.2-8.2) |
| 40-44 | 12.0 (9.4-14.5) | 6.8 (3.4-10.2) |
| 45-49 | 9.6 (7.4-11.8) | 6.3 (3.2-9.4) |
| 50-54 | 10.5 (8.1-12.8) | 7.0 (3.7-10.2) |
| 55-59 | 7.0 (5.0-9.0) | 5.3 (2.5-8.1) |
| 60-64 | 4.4 (2.5-6.2) | 2.1 (-0.4-4.6) |
| Household income |  |  |
| 1st (lowest) quartile | 7.3 (5.6-8.9) | 9.0 (5.9-12.1) |
| 2nd quartile | 8.9 (6.9-11.0) | 6.6 (3.7-9.5) |
| 3th quartile | 12.3 (9.6-15.0) | 4.8 (1.9-7.7) |
| 4th (highest) quartile | 12.1 (8.6-15.6) | 3.1 (0.1-6.1) |
| Education attainment |  |  |
| Primary school and lower | 8.3 (6.7-9.9) | 2.0 (-0.7-4.7) |
| Junior school | 8.4 (7.2-9.5) | 6.2 (4.6-7.8) |
| Senior school and higher | 10.8 (5.7-15.8) | 4.8 (1.7-7.9) |
| **Cervical cancer screening** |  |  |
| Age group |  |  |
| 35-39 | 10.0 (7.0-13.1) | 5.3 (1.7-9.0) |
| 40-44 | 8.9 (6.5-11.3) | 9.0 (6.1-12.0) |
| 45-49 | 8.5 (6.5-10.5) | 6.9 (4.2-9.6) |
| 50-54 | 8.3 (6.2-10.5) | 7.5 (4.8-10.3) |
| 55-59 | 6.1 (4.2-8.0) | 4.1 (1.7-6.6) |
| 60-64 | 4.5 (2.8-6.3) | 0.2 (-2.1-2.6) |
| Household income |  |  |
| 1st (lowest) quartile | 6.1 (4.5-7.8) | 8.9 (6.0-11.7) |
| 2nd quartile | 9.3 (7.4-11.1) | 6.8 (4.2-9.4) |
| 3th quartile | 8.4 (6.1-10.8) | 5.6 (3.1-8.2) |
| 4th (highest) quartile | 9.7 (6.5-12.9) | 1.4 (1.2-3.9) |
| Education attainment |  |  |
| Primary school and lower | 6.0 (4.6-7.5) | 2.0 (0.6-4.5) |
| Junior school | 7.7 (6.6-8.8) | 6.0 (4.6-7.4) |
| Senior school and higher | 13.9 (9.3-18.6) | 5.4 (2.6-8.1) |

Note: Increase in participation rate was calculated from the difference between intervention and comparison group after propensity score matching.
